# The impact of guided self-study on knowledge and skills in Swiss pre-clinical physiotherapy students – a feasibility study protocol

**DOI:** 10.3389/fmed.2023.939228

**Published:** 2023-05-09

**Authors:** Elisabeth Schenk, Jan Taeymans, Slavko Rogan

**Affiliations:** ^1^Department of Health, Discipline of Physiotherapy, Bern University of Applied Sciences, Bern, Switzerland; ^2^Physio Zofingen, Elisabeth Schenk, Zofingen, Switzerland; ^3^Faculty of Physical Education and Physiotherapy, Vrije Universiteit Brussel, Brussels, Belgium

**Keywords:** physical therapy speciality, graduate education, self-study, higher education, learning outcome

## Abstract

Physiotherapy education in Europe must incorporate self-study units in the curriculum due to the bologna reform. Studies investigating the impact of guided self-study (G-SS) on knowledge and skills in pre-clinical Swiss physiotherapy students are scarce. This study protocol describes a prospective randomized feasibility education study that will primarily examine the feasibility of establishing G-SS using retired physiotherapists as tutors in undergraduate physiotherapy students at the Bern University of Applied Sciences, School of Health Professions. Secondary objective will be to evaluate the effectiveness of six G-SS cycles with retired physiotherapists as tutors on knowledge and skills of pre-clinical undergraduate physiotherapy students. Students of the physiotherapy degree course will be allocated into a G-SS group or control group (CG). G-SS consists of an 8-day cycle. Feasibility outcome are the fidelity of implementation that include exposure dosage and students’ responsiveness, and the degree of acceptability. Success criteria of feasibility are (1) exposure dosage calculated as the number of 90-min presentations that are conducted, and the content of cases and competences and (2) students’ responsiveness, with at least a 83% willingness to participate. Acceptability of intervention from the undergraduate students’ perspective will be evaluated by a questionnaire with open, semi-structured questions (post intervention). This study will provide new information regarding the feasibility of embedding G-SS in the curriculum and about the students’ responsiveness and their acceptability for G-SS.

Study protocol version 1

**Trial registration:** German Register of Clinical Studies (DKRS: DRKS00015518).

## Introduction

As a part of the Bologna process, higher education institutions across the European Union were required to write their study programs and modules in terms of learning outcomes to enable the transition to the” student-centered approach (SCA)” of the European Credit Transfer System (ECTS) (1). Learning outcomes define what a student is expected to know and/or is able to demonstrate after successful completion of the degree program (1). Learning outcomes present a shift from traditional learning models that focus on inputs such as content and teaching hours towards SCA that focuses on outputs in terms of student competences (1). ECTS was introduced to provide a better representation of the students’ workload for learning and to achieve the learning outcome, respectively. The workload included work in- and outside the classroom e.g., frontal teaching, exams, self-study (SS) and for example preparation and follow-up of courses and exams expressed students time investment, whereby one credit corresponds to a workload of 25–30 h (2).

The first bachelor’s degree course (BSc) in physiotherapy at the Bern University of Applied Sciences, School of Health Professions (BFH-SHP) was launched in September 2006, with a total amount of 180 ECTS. The BFH-SHP guidelines recommend a workload ratio of 40% contact study (e.g., frontal teaching, seminars, workshops) to 60% of time for self-study (3) during a module. A module can be described as a self-contained, structured thematic unit, the scope of which is defined in learning outcomes with specified assessment criteria and ECTS credits (4).

Self-reliant learning is one of the most important components that needs to be developed while studying (4). This is described in the fifth category of the Dublin Descriptors – “learning skills” – and is to be promoted during contact studies as well as during self-study. During contact study, students are encouraged to engage in dialogue, reflection and self-critical action through adequate forms of knowledge transfer (e.g., frontal teaching, seminars, and workshops) and elaboration and the assignments. The lecturers offer students the opportunity to work on tasks independently and to provide direct feedback (4).

Landwehr and Mueller (5) and Rogan (6) described three types of self-study. The first type is free self-study (F-SS). In F-SS students set their goals, specific topics and content and develop them voluntarily according to their own interests. The second type is called individual self-study (I-SS). In I-SS self-study the students work on in greater depth, without learning outcome and work orders from lectures. The third form is guided self-study (G-SS), in which students develop knowledge, skills and competences primarily on their own but with some support of lecturers who will behave more as “coaches”. During the processing of tasks, students are supported by the lecturers. Lecturers give feedback about the process and results. The lecturers define suitable tasks that enable the students to achieve the competences that are to be developed during guided self-study. These tasks are independent respectively not linked to the learning outcomes of the contact study.

F-SS and G-SS were scheduled in the BSc physiotherapy curriculum. Students may use the F-SS to prepare for lectures (contact study) or for exam preparation or to write an essay or work on a group project or may decide themselves on which module content they want to work and learn (3). During G-SS physiotherapy students themselves may decide what they want to work on and learn under the supervision of a university lecturer (3). The university lecturer is available during this time for questions or to demonstrate or adjust practical examination and treatment techniques.

A critical stage for self-study is the initial study phase: in comparison to school, students at undergraduate level are now expected to be more independent, and usually learn more and faster (7). Harvey et al. (8) postulated that the experience of being a first-year student who is “one of all,” and was not considered as an individual, is one of the main issues of students learning. Yorke (9) suggested that the focus of the first year of study should be on addressing individual student needs, rather than viewing first-year students as a potentially problematic group of students. To support students in this phase, a well-designed concept of G-SS is needed.

Schmidt et al. (10) stated that the curriculum content plays a role in how many students graduate and how fast they graduate. In their study, they collected from 14,000 medical students study duration and graduation rate. Medical students were enrolled in eight Dutch Universities between 1989 and 1998. They were able to demonstrate that the more time available for self-study, the higher the number of students who graduated 5–8 years later. The number of classroom lessons was negatively related to graduation rate. This means, the more classroom lessons were planed and were given, the lower the graduation rate. Furthermore, a higher degree of self-study time was associated with a shorter duration of study. Lessons had a negative impact on study duration. The number of tutorial sessions and internships were not related to the outcome variables, but both were moderately negatively related to the number of classes: the more classes in a curriculum, the fewer tutorials and internships. The literature describes that a curriculum that is prioritizing small group instruction rather than classroom teaching as the primary method of instruction generally, have higher graduation rates than other types of curricula (11, 12). G-SS can focus on small groups or individual work.

### Guided self-study in higher education

Landwehr and Müller (5) postulated a five-phase concept for G-SS, in which university lecturers may guide students. Phase 1: Initiation - students will be assigned to groups and will receive a clinical case which is aligned with the curriculum content; Phase 2: Realization - students prepare the case in small groups; Phase 3: Presentation - students present their results to the lecturers and peers in a moderated plenum session; Phase 4: Evaluation – students perform a self-evaluation. Phase 5: University lecturer gives feedback to the students.

Rogan (6) described a theoretical construct of how a G-SS should be designed to change knowledge and skills among undergraduate physiotherapy students. Based on this theoretical construct, Rogan et al. (13, 14) have conducted two G-SS feasibility studies with university lecturers as tutors.

Both G-SS feasibility studies evaluated the fidelity of implementation as exposure (e.g., time duration of phases 3 to 5), students responsiveness and program differentiation. In addition, changes in knowledge and skills were assessed after a total of six G-SS cycles in first-semester (13) and after three G-SS cycles in the third semester (14) respectively. Both studies demonstrated that the G-SS must be scheduled in periods with a low workload, to increase the students’ responsiveness to G-SS. Furthermore, it could be illustrated that students who performed all six G-SS cycles (first semester) and all three G-SS cycles (third semester) showed a higher likelihood to pass written and practical exams as compared to students in the control group (F-SS: 1. Semester; *n* = 4 out of 25, and 3. Semester; *n* = 2 out of 26). Furthermore, six G-SS cycles resulted in a significant chance in knowledge in the G-SS group compared to the control group. No differences were determined after three G-SS sessions.

This form of teaching seems to have the potential that examination and treatment techniques can be learned by students from all health professions and medicine in G-SS. However, resources of lecturers are not endless. For this reason, retired physiotherapists will be used as tutors in the upcoming study. On the one hand, they have many years of experience and on the other hand, several students can be supervised in the same period. Due to the lack of empirical evidence on the recruitment rate and adherence to the program by retired physiotherapists, a feasibility study was first conducted.

This upcoming feasibility study would like to modify the existing study design as follows: 1. The G-SS should be scheduled in the timetable when the workload is below 40 h per week. 2. Different retired physiotherapists should be assigned as tutors to each group. The tutors support the students with their longstanding expertise and prepare the students for patient examination and their clinical work under complex conditions. This is intended to create a first link between knowledge acquired so far and its application in practice. 3. Six G-SS cycles should be carried out in the first semester (September 2019 to January 2020) to prepare students for clinical work.

### Aim

The primary aim of this feasibility study will be to evaluate the feasibility of the implementation of G-SS using retired physiotherapists as tutors in an undergraduate physiotherapy educational curriculum in terms of time of task, students’ responsiveness and students’ acceptability of G-SS. Secondary aim will be to investigate the impact of six G-SS cycles on changes in knowledge and skills in pre-clinical physiotherapy students at the BFH-SHP in terms of exam scores. The research question for this study is “What is the impact of retired physiotherapists tutored G-SS versus F-SS in terms of time of task, students’ responsiveness and students’ acceptability of G-SS as well as learning outcome on undergraduate physiotherapy students of BFH-SHP participating in the module basics of physiotherapeutic patient examination?”

## Methods

### Study setting

**Figure 1:**
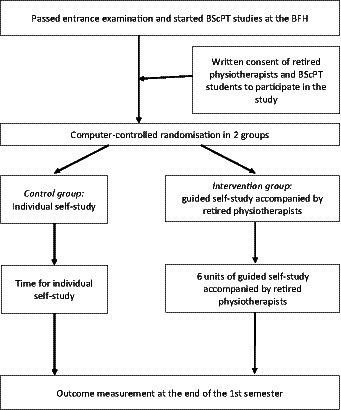
Flow of the study design.

This study is a part of a study project entitled “Retired PhysioTherapists’Tutor Supported Learning” (RePTusule). The aim of the RePTusule project is to evaluate the effects of G-SS by retired physiotherapists on learning outcomes (practical knowledge) as well as the age images of the physiotherapy students and on the physical performance and cognitive capacity of the retired tutors. This study project will start with this feasibility study. The main study will be conducted after finalization of this feasibility study, which was designed as a prospective, single-center, two-armed, randomized-group controlled educational feasibility study, conducted at the BFH-SHP (Figure 1). A feasibility study will be used prior to the main study to depict important parameters required to design the main study (15). This study will enrol undergraduate first semester physiotherapy students from the BFH-SHP and retired physiotherapists in the role of G-SS tutors.

This study protocol on G-SS is based on the SPIRIT statement (16). The overall project has been approved by the cantonal ethics commission of Bern (2018–01683) and registered in the German Register of Clinical Studies (DKRS: DRKS00015518). The participation will be voluntary. Physiotherapy students can withdraw from this feasibility study at any time. Those who withdraw voluntary from the study will follow the normal curriculum. Their data will not be used for the analysis. Students will be informed both oral and written about the study design and they will sign a consent form agreeing to participate in the study.

### Participants

The objective is to recruit a minimum of 30 first-semester physiotherapy students from the BFH-SHP (17). This *a priori* set recruitment rate will be used as a criterion of success to evaluate the feasibility and the timeframe of recruitment for the upcoming fully randomized controlled higher-education study (3). The participants are young healthy adults, are enrolled at the BFH-SHP and affiliated with the bachelor physiotherapy degree cohort. Students who do not intend to participate will be excluded from the recruitment process.

The retired physiotherapists will be recruited by means of an advertisement in the official journal “physioactive” of the Swiss Physiotherapy Association and by asking colleagues. They will be informed in writing and orally about the course of the study and their participation in the study.

### Eligibility criteria

Inclusion criteria for the students are: (1) young healthy physiotherapy students, (2) enrolled at the undergraduate physiotherapy degree course 2019 (first semester) at the BFH-SHP, and (3) signed informed consent. Exclusion criteria are: (1) physiotherapy students from undergraduate physiotherapy course 2018 who needed to repeat the first semester, (2) students from other BFH-SHP degree courses, and (3) physiotherapy students from other institutions.

Inclusion criteria for retired physiotherapists are: (1) retired or still working with patients for a maximum of 10% and (2) healthy. Exclusion criteria are: (1) work more than 10% on patients or in another profession, (2) have been employed in teaching, (3) have previously supervised student interns, and (4) have cognitive impairments.

### Intervention program

A total of six G-SS cycles with six different cases will be conducted in the first semester. The intervention program aimed to investigate feasibility and impact of six G-SS sessions on changes in knowledge and skills in pre-clinical physiotherapy students at the BFH-SHP. Students of the G-SS will be divided into five groups of five to six students each. They will work on a clinical case in an 8-day cycle. The structure of the eight-day cycle will be based on Rogan et al. (3, 6, 13, 14, 18). Phase 1, day 1: students of the G-SS and the retired physiotherapists will receive on day 1 (i.e., 1 week prior to the presentation day) via email a clinical case for processing. Phase 2, day 2–7: students will be guided by their tutors during the one-week preparatory phase. Students will have the possibility to arrange two online meeting sessions with their tutor. In these meetings, questions can be answered, and the current status and stumbling blocks can be addressed and solved. Phase 3–5, day 8: students will present their findings and solutions of their work orally and practically. In a moderated plenum session students will conclude the working process and learning process, any questions and optimization steps are clarified. Feedback will be given to the students by the tutors.

The first semester includes topics and learning objectives from the module “Basics of physiotherapeutic Patient Examination.” The topics of the first semester are: basic movement, testing coordination, manual muscle strength tests, massage techniques, movements and joint position, muscle activities, palpation, passive and active angular joint examination, statics and constitution, translational joint examination and tests for muscle flexibility. The G-SS will be scheduled in the school timetable with a total workload of <40 h per week. The case topics are listed in Table 1.

**Table 1:** Topic of the cases during the G-SS cycles in the first semester.

G-SS cycle	Topic
1.	Massage technique
2.	Joint measurement, differentiation of musculature
3.	Gait analysis
4.	Joint measurement with goniometer and app for comparison
5.	Hip joint region: Examine neighboring joints
6.	Case study in context of Clinical Reasoning

### Control condition

F-SS will be scheduled during this period. Students in the control group will be given the same amount of time in the timetable for F-SS. There is no supervision nor tutoring and students will not receive a clinical case and will receive no information on the direction of what they can do.

#### Achievement characteristics

The *a priori* set criteria of success for feasibility and preliminary data for changes in knowledge and skills will be evaluated at the end of the first semester (February 2020).

#### Primary achievement goal

Criteria of success will be operationalized as “fidelity of implementation” (19), such as time of task, students’ responsiveness, and the acceptability of the intervention from the students’ perspective and the recruitment of retired physiotherapists as tutors. “Fidelity of implementation” has been described as to which level the intervention is implemented as intended by those who developed it (19, 20).

#### Measures

##### The fidelity of implementation will be assessed for

1. time of task as (i) participation at all six presentation days (i.e., days 8 of the cycles; phase 3–5), and (ii) the duration of the session on day 8 is limited to maximum 90 min.
2. students’ responsiveness will be recorded by the retired physiotherapists in an attendance list on the presentation day eight. Criteria of success will be defined as at least 83% of the physiotherapy students of the G-SS taking part in five out of six presentation days (i.e., day 8 of the cycles; phase 3–5).

##### Acceptability of intervention from students’ perspective

Acceptability of G-SS will be assessed from the physiotherapy students using a questionnaire with open, semi-structured questions at the end of day 8 of the sixth G-SS cycle (post intervention). The interview protocol will obtain the students’ opinion and experiences of the G-SS.

##### Recruitment of retired physiotherapists

Retired physiotherapist should work as tutors with groups of six students. A total of five tutors will be recruited. Criteria of success will be set as 100% if five retired physiotherapists can be recruited.

#### Secondary achievement goals

These include the learning outcomes, satisfaction, self-efficacy, and students’ images of old age. Physical performance level of the retired physiotherapists and the G-SS implementation into the curriculum.

### Outcome measures

1. Students’ learning outcome

- Students’ learning outcome could be defined as of what a learner is expected to know, understand or be able to do at the end of a period of learning (21). Learning outcome will be measured based on the semester exams (ordinal scaled) including written multiple-choice questions (MCQs) and an Objective Structured Clinical Examination (OSCE). The written exams consist of 87 single best answer MCQs. The OSCE consist of eight 8-min stations. The maximum score will be 87 and 64 points for the MCQs and OSCE, respectively. The exam will be passed if 60% of the total score is reached.
- Student’s satisfaction will be assessed with the student learning satisfaction questionnaire (22).
- Self-efficacy will be assessed using the self-efficacy learning (SEL) questionnaire (23).

1. Level of retired physiotherapists’ physical and cognitive performance. Physical performance capacity will be evaluated using the Short-Physical-Performance-Battery-Test while evaluation of the cognitive performance will be assessed using the Trail-Making A and B test before the start of the tutorship after the end of the first and second semester, respectively.
2. Level of age images of physiotherapy students are assessed before the G-SS and F-SS lessons at the end of first and second semester, respectively. Age images will be assessed with the age images questionnaire (24).
3. Level of G-SS curriculum implementation: the integration of G-SS into the existing curriculum will take place after evaluation of this planned feasibility study (i.e., after the second semester).

### Potential confounders

Education takes place in different social environments (e.g., classroom, mentoring where lecturers have an impact). Students may have different expectations regarding learning support by lecturers. As a result, this may affect learning gains during the first semester. In this case, classroom instruction mentoring will not be carried out in the first semester and lectures will take place according to the traditional teaching method. There are neither Moodle exercises nor preparatory assignments in the sense of a blended learning approach scheduled during the first semester.

### Covariates

Age, gender, local geographic matching, duration of school attendance and student cohort will be identified and recorded as possible covariates.

### Sample size

At the BFH-SHD bachelor’s degree course are always 102 bachelor students enrolled that will be allocated into two groups. We want to recruit five retired physiotherapists. Assuming the intra-cluster correlation coefficient (25) of 0.05 a design effect of 5.25 is given. Sample size is for this study 111.3 (612/5.25).

### Allocation concealment

The bachelor physiotherapy degree course regularly allocates students to groups A, B, C or D at the start of every semester. An independent employee of the BFH-SHP carries out the allocation of students by means of a computer-generated random number (Excel, Microsoft). No other member of the institute or the research team has any influence on the randomization process or access to this data. The independent employee keeps the original random number sequences in an inaccessible third location (26). Groups A and B and Groups C and D are together during practical courses to ensure small group sizes. This classification is determined in advance of the start of every semester independent of the investigation by the responsible persons of the educational program. For this planned feasibility study, randomization will be group-based. An independent researcher will assign groups A/B and groups C/D to the G-SS F-SS groups by tossing a coin.

### Data collection methods

The first collection of data by means of surveys is scheduled for the start of the project (T0). Thereafter, the same surveys will be filled out by the students participating in this planned feasibility study after the fall semester (T1) and after the spring semester (T2). Before-and-after learning effects will be shown in the intervention group and the control group as well as between the groups are presenting using parametric methods. If a retired physical therapist, or a physiotherapy student, does not wish to continue participating in this study, that person will, upon request immediately removed from the study program. The date of discontinuation will be noted and placed in the files or records. The data will be anonymized after the analysis.

### Statistics

Because this study is a feasibility study, testing intervention effects is not a key part of this study design (18). Baseline descriptive statistics will be used to describe G-SS and F-SS group characteristics and to illustrate the feasibility goals. The independent variable will be G-SS. The dependent variable will be the final grades of the MCQs and OSCE. Preliminary group differences at the end of the first semester will be analyzed with the Mann–Whitney-U-Test. The significance level will be set *a priori* to *p* = 0.05. An intention-to-treat analysis will always be the primary analysis of a randomized trial in higher education (17). An ITT will be carried out if physiotherapy students do not adhere to the G-SS protocol.

The findings of the satisfaction and self-efficacy items will be summarized visually in a table and presented as response distributions (number, percentage). Satisfaction analysis will involve the summation satisfaction scale scores, with 0.80 as the proposed threshold for defining usability and acceptability, counting only positive endorsements (4 = agree, 5 = strongly agree). Qualitative data from the semi-structured interview will be analyzed thematically.

## Discussion

The strength of this planned feasibility study is, that we will be able to incorporate findings from previous studies with a similar structure (13, 14). In contrast to the two previous studies, students will not be guided by only one teacher who was tutoring all groups, but by six retired physiotherapists each tutoring a single group. There is a need to examine the changes in knowledge and (patient examination) skills achieved through G-SS. Evidence on feasibility, students’ responsiveness to the G-SS program, acceptability, and the impact of G-SS on knowledge gains and skills improvements is needed.

It is assumed that volunteers of the G-SS will demonstrate greater improvement in knowledge and skills as compared to their peers participating in F-SS. A previous study has demonstrated that G-SS may influence knowledge changes and skills changes in undergraduate physiotherapy students at the BFH-SHP (13). This planned feasibility study has the possibility to further evaluate these issues and deliver new information regarding the embedding of the G-SS in the curriculum, about the students’ responsiveness and acceptability of the students toward the G-SS program.

Studies with randomized controlled designs in higher education are not used very often because of the blunt study design that ignores context and experience of learning and teaching, and these types of study tends to ignore the complexities in the higher education landmark and present simplistic universal laws of cause and effect (27). Nevertheless, a randomized study design reduces the allocation bias resulting from baseline variables that may negatively affect the results (18). Furthermore, a randomized study design can be used in an educational setting if the study design uses standardized intervention methods (28). Concealment is impossible as students talk to each other. Hence, it remains an issue and a study limitation.

This feasibility study will utilize a randomized controlled trial design to reduce bias and to strengthen the preliminary results. This study will be conducted in real-life situation and will contribute to real-world higher-education settings. Higher education research usually uses non-experimental and less representative research methods that prove unsuitable for use in education policy (29).

## Ethics statement

Ethical approval was obtained from the Kantonalen Ethikkommission Bern, Switzerland (2018-01683).

## Author contributions

This research project was developed by SR and JT. SR performed the data collection, carried out the statistical analysis, and wrote the manuscript. JT and ES edited the manuscript. All authors contributed to the article and approved the submitted version.

## Funding

This feasibility study was funded by the Foundation Sana and Gesundheitsförderung Schweiz.

## Conflict of interest

The authors declare that the research was conducted in the absence of any commercial or financial relationships that could be construed as a potential conflict of interest.

## Publisher’s note

All claims expressed in this article are solely those of the authors and do not necessarily represent those of their affiliated organizations, or those of the publisher, the editors and the reviewers. Any product that may be evaluated in this article, or claim that may be made by its manufacturer, is not guaranteed or endorsed by the publisher.

## Abbreviations

BFH-SHP: Bern University of Applied Sciences – School of Health Professions
BSc: Bachelor of Science
CG: Control group
CL: Contact lecture
ECTS: European Credit Transfer System
F-SS: Free self-study
G-SS: Guided self-study
IG: Intervention group
MCQs: Multiple-choice questions
OSCE: Objective structured clinical examination
rP: Retired Physiotherapist
SPSS: Statistical Package for Social Sciences
